# Biological Role of Cobalt(II), Copper(II) and Nickel(II) Metal Ions on the Antibacterial Properties of Some Nicotinoyl-Hydrazine Derived Compounds

**DOI:** 10.1155/MBD.1997.69

**Published:** 1997

**Authors:** Zahid H. Chohan, Syed K. A. Sherazi

**Affiliations:** 1 Department of Chemistry Islamia University Bahawalpur Pakistan; 2 Department of Chemistry University of Aberdeen Meston Walk Scotland Old Aberdeen AB9 2UE UK

## Abstract

Several cobalt(II), copper(II) and nickel(II) complexes of nicotinoylhydrazine-derived compounds were prepared and characterised by physical, spectral and analytical data. These compounds and their complexes have proven to be antibacterial. The screening data show the metal complexes to be more potential/bactericidal than the uncomplexed compounds against one or more bacterial species.


### BIOLOGICAL ROLE OF COBALT(II), COPPER(II) AND NICKEL(II) METAL IONS ON THE ANTIBACTERIAL PROPERTIES OF SOME NICOTINOYL-HYDRAZINE DERIVED COMPOUNDS

Zahid H. Chohan* and Syed K. A. Sherazi

Department of Chemistry, Islamia University, Bahawalpur, Pakistan

### ABSTRACT
Several cobalt(II), copper(II) and nickel(II) complexes of nicotinoylhydrazine-derived compounds were prepared and characterised by physical, spectral and analytical data. These compounds and their complexes have proven to be antibacterial. The screening data show the metal complexes to be more potential/bactericidal than the uncomplexed compounds against one or more bacterial species.

### INTRODUCTION
The increasing interest in the chemistry of hydrazines and hydrazones because of their potential biological applications[1-4] have drawn a considerable attention during the past few years. Many reports[5-9] have indicated that biologically active compounds/drugs become more carcinostatic and bacteriostatic upon coordination with the metal ions. As a part of our ongoing research programme in elaborating more this pronounced biological role of metal ions we have previously reported and as a further contribution, we have prepared some more biologically active nicotinoylhydrazine derived compounds $L_1$-$L_4$ (Fig. 1) and their Co(II), Cu(II) and Ni(II) complexes and hence, wish to report the role of metal ions on their biological activity. These synthesised ligands and their complexes have been characterised on the basis of conductance and magnetic measurements, elemental analysis and $^1$H-NMR, IR and electronic spectral data. These ligands and their complexes have been screened for their possible antibacterial activity against bacterial strains of *Escherichia coli, Pseudomonas aeruginosa* and *Staphylococcus aureus.* The antibacterial activity data of the ligands are known to be substantially increased upon complexation against one or more bacterial species.

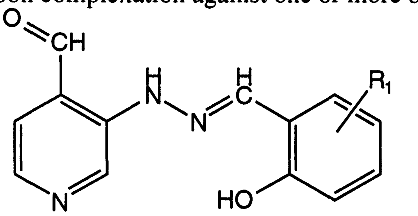

$L_1$: $R_1$ = H
$L_2$: $R_1$ = 5-$CH_3$
$L_3$: $R_1$ = 3-$OCH_3$
$L_4$: $R_1$ = 5-Cl

Figure 1: Structure of the ligands

### EXPERIMENTAL
### Material and Methods
All chemicals used in this work were of reagent grade. Co(II), Cu(II), and Ni(II) were used as their chlorides. Conductance and magnetic measurements were made on a YSI model-32 conductivity bridge and Gouy balance respectively. Infrared spectra were recorded on a $R_{10}$ Hitachi spectrophotometer. $^1$H-NMR spectra were obtained on a $R_{10}$ Perkin-Elmer spectrometer. Electronic spectra were studied on a Hitachi double-beam U-2000 model spectrophotometer using glass cells of 1 cm thickness. Elemental analysis of C, H and N were determined on a Coleman automatic analyser. All melting points were taken on a Gallenkamp melting point apparatus and were uncorrected.

Antibacterial studies were carried out with the help of the Microbiology Laboratory, Department of Pathology, Qaid-e-Azam Medical College, Bahawalpur. These studies were done on wild pathogenic bacterial species collected from the urine and blood samples of infected patients admitted in Bahawal Victoria Hospital, Bahawalpur.

* Author for correspondence: Department of Chemistry, University of Aberdeen, Meston Walk, Old Aberdeen AB9 2UE, Scotland, UK

**Preparation of Ligands**

Ligands $L_1$-$L_4$ (Fig 1) were prepared according to the method reported[10] earlier.

**Preparation of Metal Complexes**

To a hot n-butanol solution(30 mL) of the ligand (0.004 mol) was added an ethanolic solution (15 mL) of the respective metal(II) chloride (0.002 mol). The mixture was refluxed for 2 h. The resulting solution was cooled, filtered and reduced to 20 mL with an evaporator.The concentrated solution so obtained was left overnight at room temperature resulting in the formation of a solid product. The solid product was filtered, washed with n-butanol (2x10 mL) and dried. Crystallisation from aqueous n-butanol(50%) gave 1 (82%), 2 (80%), 3 (78%), 4 (79%), 5 (80%), 6 (81%), 7 (77%), 8 (81%), 9 (82%), 10 (75%), 11 (77%) and 12 (79%).

**Antibacterial Studies**

Antibacterial activity of the prepared ligands and their complexes was tested against bacterial species, obtained from the different patients carrying these bacteria, *Escherichia coli, Pseudomonas aeruginosa* and *Staphylococcus aureus* using the paper disc diffusion method.

**Preparation of Discs**

A ligand/complex (30 g) DMF solution (0.01 mL) was applied on a paper disc prepared from blotting paper (3 mm size) with the help of a micropipett. The discs were left in an incubator for 48 h at 37°C and then applied on bacteria grown agar plates.

**Preparation of Agar Plates**

For this purpose minimal agar was used for the growth of specific bacterial species. For Staphylococcus species, a blood agar base with low pH was used. The blood agar base (40 g) was first suspended in cold distilled water (1L) and heated to boiling. It was then sterilised at 120°C for 15 minutes and later allowed to cool at 50°C. Then 5% sterile defibrinated cow blood was added to it and the mixture was poured into previously washed and sterilised Petri dishes which were stored at 4°C for inoculation. For the preparation of agar plates for Escherichia coli and Pseudomonas species, Mac Conkey agar (50 g), obtained from Merck Chemical Company, was suspended in freshly distilled water (1L). It was allowed to soak for 15 minutes and then boiled with constant shaking in a water bath until the agar was completely dissolved. The mixture was autoclaved for 15 minutes at 120°C and then poured into previously washed and sterilised Petri dishes and stored at 4°C for inoculation.

**Procedure of Inoculation**

Inoculation was done with the help of a platinum wire loop which was made red hot on a flame, allowed to cool in air and then used for the application of previously described bacterial strains. The preculture was first prepared in 2 mL of a nutrient broth by selecting a suitable bacterial colony and later on transfered to a nutrient broth which was incubated for 2 h at 37°C. Then 500 L of the culture was spread on the specific agar plates, which was incubated for 24 h at 37°C.

**Application of Disc**

A sterilised forecep was used for the application of paper disc on the already inoculated agar plates. When the disc was applied, it was then incubated at 37°C for 24 h. The the zone of inhibition in diameter was measured.

### RESULTS AND DISCUSSION

The structural determination of ligands was done on the basis of their IR, $^1$H-NMR and elemental analysis data (Table 1). It is known that benzoyl or nicotinoyl hydrazines exhibit keto-enol tautomerism and as such they can exist in one of these forms. If the ligands exist in the keto form the (NH) and (C=O) absorption bands will appear in the infrared spectra, whereas the absence of these two absorptions may be indicative of enol form. All these ligands show a broad band at 3440 cm$^{-1}$ and a medium intensity band in the range 3280-3390 cm$^{-1}$ attributed to (OH) and (NH). A strong band at 1670 cm$^{-1}$ is assigned to (C=O) absorption. An intense band in the range 1645-1650 cm$^{-1}$ is assigned[21,22] to (C=N). The presence of these bands thus confirm that the ligands exist in the keto form. The $^1$H-NMR spectra of the ligands (Table 1) also display signals assignable to the azomethine (CH=N) and amide (NH) protons and all other expected protons. Also, the microanalytical data (Table 1) of the ligands was found to be in agreement with the molecular structures of the title ligands.

All the metal complexes 1-12 of these ligands were prepared by the stoichiometric reaction of metal(II) chloride and the respective ligand. The result of the elemental analysis (Table 2) indicate that all the complexes have 1:2 (metal:ligand) stoichiometry. All the complexes are coloured, amorphous and insoluble in most organic solvents. The complexes are soluble in DMF and DMSO. The conductance values in DMF are found in the range 18-25 ohm$^{-1}$cm$^2$mol$^{-1}$ which indicate that all the complexes behave as non-electrolytes[23,24].

The magnetic susceptibility measurements (Table 2) for the solid complexes at room temperature indicate that the effective magnetic moment ($\hat{E}_{eff}$) for all the complexes lie well within the range for their observed geometries[25,26]. The $\hat{E}_{eff}$ for Co(II) complexes fall in the range 4.16-4.52 B.M. expected to contain odd number of electron (d$^7$-system). The $\hat{E}_{eff}$ values(1.53-1.57 B.M.) for Cu(II) ion are indicative of one unpaired electron per Cu(II) ion and two unpaired electrons per Ni(II) ion ($\hat{E}_{eff}$ = 2.78-2.95 B.M) suggesting[27-29] to lie within the range consistent to their spin-free octahedral geometry for Co(II) and Ni(II) complexes and a distorted octahedral geometry for Cu(II) complexes.

**Table 2** Physical, Spectral and Analytical Data of Metal(II) Complexes

| No | Mol.Formula | M.P. (°C) | B.M. ($\mu_{eff}$) | IR ($cm^{-1}$) | $\lambda_{max}$($cm^{-1}$) | Cal(Found)% C | H | N |
|---|---|---|---|---|---|---|---|---|
| 1 | $[Co(L_1)_2]Cl_2$ $C_{26}H_{16}Cl_2CoN_6O_4$ | 158-160 | 4.16 | 3425,3358,2819,2010, 1665,1640,1380,1290, 1010,918,720,510,445 | 19525,17115, 8555 | 51.52 (51.84) | 2.63 (2.55) | 13.85 (13.67) |
| 2 | $[Co(L_2)_2]Cl_2$ $C_{26}H_{20}Cl_2CoN_6O_4$ | 149-151 | 4.52 | 3430,3365,2819,2010, 1662,1635,1380,1290, 1010,918,720,515,450 | 20170,17260, 8450 | 53.03 (52.91) | 3.15 (3.56) | 13.24 (13.15) |
| 3 | $[Co(L_3)_2]Cl_2$ $C_{28}H_{20}Cl_2CoN_6O_6$ | 155-157 | 4.33 | 3435,3360,2819,2714, 2010,1660,1635,1380, 1290,1118,1010,918, 720,525,445 | 19735,17222, 8550 | 50.55 (50.68) | 3.00 (2.88) | 12.62 (12.86) |
| 4 | $[Co(L_4)_2]Cl_2$ $C_{26}H_{14}Cl_3CoN_6O_4$ | 163-165 | 4.28 | 3432,3355,2819,2010, 1660,1635,1440,1385, 1290,1118,1011,918, 525,450 | 19810,17220, 8465 | 48.82 (48.76) | 2.18 (2.06) | 13.13 (13.34) |
| 5 | $[Cu(L_1)_2]Cl_2$ $C_{26}H_{16}Cl_2CuN_6O_4$ | 187-189 | 1.57 | 3430,3365,2819,2010, 1665,1630,1385,1292, 1118,1010,918,525, 445 | 30675,22150, 15222 | 51.13 (51.44) | 2.61 (2.84) | 13.75 (13.69) |
| 6 | $[Cu(L_2)_2]Cl_2$ $C_{28}H_{20}Cl_2CuN_6O_4$ | 178-180 | 1.53 | 3425,3350,2819,2010, 1665,1645,1380,1295, 1010,918,515,445 | 29515,22580, 14255 | 52.64 (52.56) | 3.13 (3.02) | 13.15 (13.37) |
| 7 | $[Cu(L_3)_2]Cl_2$ $C_{28}H_{20}Cl_2CuN_6O_6$ | 181-182 | 1.55 | 3435,3350,2819,2010, 1660,1645,1385,1292, 1010,918,725,525,450 | 30270,22220, 15135 | 50.13 (50.48) | 2.98 (2.88) | 12.52 (12.46) |
| 8 | $[Cu(L_4)_2]Cl_2$ $C_{26}H_{14}Cl_2CuN_6O_4$ | 190-192 | 1.54 | 3430,3358,2819,2010, 1662,1635,1380,1290, 1010,918,725,510,445 | 29715,22250, 14250 | 48.47 (48.51) | 2.17 (2.10) | 13.04 (13.29) |
| 9 | $[Ni(L_1)_2]Cl_2$ $C_{26}H_{16}Cl_2NiN_6O_4$ | 128-130 | 2.78 | 3425,3355,2819,2010, 1660,1635,1380,1295, 1010,918,720,515,445 | 26280,15465, 9580 | 51.54 (51.71) | 2.64 (2.48) | 13.84 (13.77) |
| 10 | $[Ni(L_2)_2]Cl_2$ $C_{26}H_{16}Cl_2NiN_6O_4$ | 123-125 | 2.57 | 3435,3358,2819,2015, 1665,1635,1380,1295, 1118,1010,918,725, 515,450 | 26110,15570, 9620 | 53.05 (53.18) | 3.15 (3.57) | 13.25 (13.08) |
| 11 | $[Ni(L_3)_2]Cl_2$ $C_{28}H_{20}Cl_2NiN_6O_6$ | 117-120 | 2.82 | 3425,3365,2819,2010, 1665,1645,1380,1295, 1010,918,525,445 | 26225,15490, 9615 | 50.50 (50.61) | 3.00 (2.78) | 12.61 (12.40) |
| 12 | $[Ni(L_4)_2]Cl_2$ $C_{26}H_{14}Cl_3NiN_6O_4$ | 132-135 | 2.95 | 3430,3355,2819,2010, 1662,1630,1385,1290, 1118,1010,918,725, 510,445 | 26220,15555, 9595 | 48.84 (48.90) | 2.18 (2.31) | 13.1 (13.06) |

The bonding of the ligands to the metal ions was determined by comparing the infrared spectra of the free ligand and the spectra of their metal complexes. In all the complexes the band corresponding to (NH) is retained. The (C=N) band shows a low frequency shift in the spectra of the complexes, indicating that the nitrogen of the azomethine moiety has coordinated to the metal ion. Also the disappearance of (OH) and (C=O) absorption bands and a distinct low frequency shift of these bands by 30-40 $cm^{-1}$ is suggestive of the coordination of hydroxyl and carbonyl oxygens to the metal ion. However, the appearance of two new bands observed in the spectra of the metal complexes and not observable in the spectra of the ligands within 445-450 $cm^{-1}$ and 510-525 $cm^{-1}$ assigned respectively to M-O and M-N modes confirmed the involvement of nitrogen of azomethine (C=N) and oxygen heteroatoms of (OH) and (C=O) groups in the coordination of the ligands to the metal atom.

**Table 1 Physical, Spectral and Analytical Data of the Ligands**

| No | Mol. Formula | M.P ($^oC$) | Yield (%) | IR ($cm^{-1}$) | $^1H$-NMR(ppm) | Calc (Found) % C | H | N |
|---|---|---|---|---|---|---|---|---|
| $L_1$ | $C_{13}H_{10}N_3O_2$ | 97 | 82 | 3440,3383, 2819,2010, 1672,1645, 1380,1292, 1011,918 | 6.21(s,1H,NH) 6.22-6.68(m,2H,Ph) 6.90-7.18(m,2H,-Ph) 7.34-7.42(m,2H,nicotinoyl),7.63(d,1H,-nicotinoyl),8.30(s,1H,azomethine),10.20(s,1H,CHO), 11.32(s,1H,OH) | 64.96 (65.38) | 4.16 (4.09) | 17.49 (17.11) |
| $L_2$ | $C_{14}H_{12}N_3O_2$ | 110 | 78 | 3440,3390, 2818,2010, 1650, 1380, 1291,1012, 918 | 1.89(s,3H,$CH_3$) 6.22(s,1H,NH) 6.92-7.17(m,2H,-Ph) 6.53-6.79(m,1H,-Ph) 7.33-7.45(m, 2H,nicotinoyl),7.60(d,1H,nicotinoyl),8.30(s,1H,azomethine),10.23(s,1H,CHO) 11.32(s,1H,OH) | 66.16 (65.92) | 4.72 (4.96) | 16.52 (16.18) |
| $L_3$ | $C_{14}H_{12}N_3O_3$ | 92 | 80 | 3440,3385, 2819,2010, 1648,1380, 1294,1010, 918,806 | 2.64(s,3H,$OCH_3$) 6.22(s,1H,NH) 6.71-6.83(m,2H,-Ph) 6.42-6.51(m,1H,-Ph) 7.23-7.31(m,2H, nicotinoyl),7.60(d,1H,nicotinoyl),8.30(s,1H,azomethine),10.22(s,1H,CHO) 11.32(s,1H,OH) | 62.24 (62.11) | 4.44 (4.34) | 15.54 (15.29) |
| $L_4$ | $C_{13}H_9N_3O_2Cl$ | 102 | 82 | 3440,3382, 2819,2011, 1645,1385, 1291,1010, 918 | 6.21(s,1H,NH) 6.98-7.12(m,2H,-Ph) 6.51-6.66(m,1H,-Ph) 7.32-7.48(m,2H,nicotinoyl),7.62(d,1H,nicotinoyl),8.31(s,1H,azomethine),10.20(s,1H,CHO) 11.33(s,1H,OH) | 56.86 (57.12) | 3.27 (3.08) | 15.29 (15.16) |

In the electronic spectra(Table 2) of the cobalt(II) complexes, three spin allowed transitions were observed at 8450-8555, 17115-17260 and 19520-20175 $cm^{-1}$ due to the transitions $^4T_{1g}(F) \rightarrow {}^4T_{2g}(F)(V_1)$, $^4T_{1g}(F) \rightarrow {}^4A_{2g}(F)(V_2)$ and $^4T_{1g}(F) \rightarrow {}^4T_{1g}(P)(V_3)$ respectively which were suggestive[30,31] of their octahedral geometry. The nickel(II) complexes also exhibit three typical bands around 9580-9620, 15465-15570 and 26110-26280 $cm^{-1}$ corresponding to the transitions $^3A_{2g} \rightarrow {}^3T_{2g}(V_1)$, $^3A_{2g} \rightarrow {}^3T_{1g}(F)(V_2)$ and $^3A_{2g} \rightarrow {}^3T_{2g}(F)(V_3)$ respectively assigned for octahedral field[32]. The copper(II) complexes showed a broad band around 14250-15222 $cm^{-1}$ arising from the $^2E_g \rightarrow {}^2T_{2g}$ transition in octahedral environment with distorted geometry and the other two bands around 22150-22580 and 29515-30675 $cm^{-1}$ can be attributed[33] to intra-ligand charge transfer transitions.

It is thus concluded on the basis of the above observations that all the metal(II) complexes possess an octahedral geometry in which the two ligands behaving as tridentate accommodate themselves around the central metal atom in such a way that a stable configuration of a metal chelate is formed (Fig. 2).

### Antibacterial Studies

The synthesised ligands and their metal complexes were tested for their antibacterial activity against bacterial species *Escherichia coli, Pseudomonas aeruginosa* and *Staphylococcus aureus*. The antibacterial activity of these compounds was tested at a concentration of 30 g/0.01 mL in DMF using paper disc diffusion method as described previously[10-12]. The same method was applied for assessing the activity, the results of which are reported in Table 3.

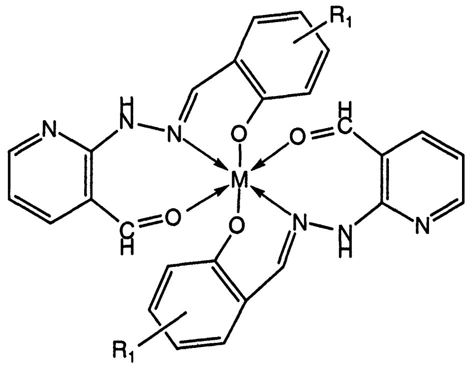

Fig 2. Proposed structure of the metal chelate

**Table 3** Antibacterial Activity Data

| Ligands/ Complexes | Microbial Species a | b | c |
|---|---|---|---|
| $L_1$ | + | ++ | - |
| $L_2$ | + | ++ | ++ |
| $L_3$ | - | + | ++ |
| $L_4$ | + | ++ | ++ |
| 1 | ++ | +++ | ++ |
| 2 | ++ | ++++ | ++ |
| 3 | ++ | +++ | +++ |
| 4 | +++ | ++ | +++ |
| 5 | ++ | +++ | ++ |
| 6 | ++ | +++ | +++ |
| 7 | +++ | +++ | ++++ |
| 8 | ++ | +++ | +++ |
| 9 | ++ | +++ | ++ |
| 10 | +++ | +++ | +++ |
| 11 | ++ | +++ | ++ |
| 12 | +++ | +++ | +++ |

a = *Escherichia coli,* b = *Pseudomonas aeruginosa,*
c = *Staphylococcus aureus*; Inhibition zone diameter (mm),
+, 6-10; ++, 10-14; +++, 14-18; ++++, 18-22

The results of these studies clearly indicate that the ligands and their complexes are all potent and biologically active against one or more testing bacterial strains. More so, the metal complexes have shown to be more antibacterial against the same testing species than the simple uncomplexed ligand. This, in turn, has confirmed our all previous studies[10-18] that the metal chelation increases the potency/biological activity of such compounds/drugs which have bactericidal characteristic properties.

### ACKNOWLEDGEMENT

The authors are gratefull to the Department of Pathology, Qaid-e-Azam Medical College, Bahawalpur for their help in undertaking the antibacterial studies.

**REFERENCES**

1 J.R.Dilworth, *Coord.Chem.Rev.*, 1976, **21**: 29

2 M.Katyal and Y.Datta,*Talanta.*, 1975, **22**: 151

3 K.Redda, L.A.Corleto and E.E.Knaus, *J.Med.Chem.*, 1979, **22**: 1079

4 J.R.Merchant and D.S.Chothia, *J.Med.Chem.*, 1970, **13**: 335

5 D.R.William, *Chem.Rev.*, 1972, **72**: 203

6 M.J.Seven and L.A.Johnson, "*Metal Binding in Medicine*", 1960, 4th Ed, Lippincott Co, Philadelphia P.A

7 D.R.Williams, "*The Metal of Life - The Solution Chemistry of Metal ions in Biological System*", 1971, Van Nostrand, London

8 M.J.Clare and J.D.Heeschele, *Bioinorg.Chem.*, 1973, **2**: 187

9 A.Furst, "*The Chemistry of Chelation in Cancer*", 1963, 3rd Ed, Springfield, Illinois

10 Z.H.Chohan and A.Rauf, *Synth.React.Inorg.Met.-Org.Chem.*, 1996, **26**: 591

11 Z.H.Chohan and M.A.Farooq, *J.Chem.Soc.Pak.*, 1995, **17**: 14

12 Z.H.Chohan and M.A.Farooq, *Pak.J.Pharmaceut.Sci.*, 1994, **7**: 45

13 Z.H.Chohan and H.Pervez, *Synth.React.Inorg.Met.-Org.Chem.*, 1993, **23**: 1061

14 Z.H.Chohan and A.Rauf, *J.Inorg.Biochem.*, 1992, **46**: 41

15 Z.H.Chohan and S.Kausar, *Chem.Pharm.Bull.*, 1993, **41**: 951

16 Z.H.Chohan and S.Kausar, *Chem.Pharm.Bull.*, 1992, **40**: 2555

17 Z.H.Chohan and F.Alam, *Pak.J.Pharmacol.*, 1991, **8**: 8

18 Z.H.Chohan, *Chem.Pharm.Bull.*, 1991, **39**: 1578

19 L.Sacconi, *J.Am.Chem.Soc.*, 1952, 74: 4503

20 K.K.Narang and A.Agarwal, *Inorg.Chim.Acta.*, 1974, **9**: 137

21 L.W.Lane and C.T.Taylor, *J.Coord.Chem.*, 1973, **2**: 295

22 A.E.Martin, *Nature*, 1950, **166**: 474

23 A.M.Shallary, M.M.Moustafa and M.M.Bekheit, *J.Inorg.Nucl.Chem.*, 1979, **41**: 267

24 W.J.Geary, *Coord.Chem.Rev.*, 1971, **7**: 81

25 M.D.Glick and R.L.Lintvedt, *Prog.Inorg.Chem.*, 1976, **21**: 233

26 C.J.Balhausen,"*An Introduction to Ligand Field*", 1962, Mc Graw Hill, New York

27 A.B.P.Lever, *Inorg.Chem.*, 1965, **4**: 763

28 V.B.Rama, D.D.Singh, P.Singh and M.Teotia., *Trans.Met.Chem.*, 1981, **6**: 36

29 E.K.Barefield, D.H.Busch and S.M.Nelson, *Quart.Rev.*, 1968, **22**: 457

30 A.D.Liehr, *J.Phys.Chem.*, 1967, **67**: 1314

31 R.L.Carlin,"*Transition Metal Chemistry*", 1965, Vol I, Marcel Decker, New York

32 D.W.Meek, R.S.Drago and T.S.Piper, *Inorg.Chem.*, 1962, **1**: 285

33 A.B.P.Lever, "*Inorganic Electronic Spectroscopy*", 1984, IInd Ed, Elsevier, New York

**Received: October 21, 1996 - Accepted: November 4, 1996 - Received in revised camera-ready format: March 7, 1997**

